# Fibular grafts in global reconstructive surgery: a bibliometric analysis

**DOI:** 10.3389/fsurg.2024.1479878

**Published:** 2024-11-18

**Authors:** Zhi Zhang, Yuezhan Li, Lin Cheng, Ying Deng, Yan Cai

**Affiliations:** ^1^ Health Management Department, The First Hospital of Hunan University of Chinese Medicine, Changsha, Hunan, China; ^2^ School of Medicine, Regenerative Medicine Institute, University of Galway, Galway, Ireland; ^3^ Graduate School, Hunan University of Chinese Medicine, Changsha, Hunan, China; ^4^ Spinal Surgery Department, Hunan Provincial Hospital of Integrated Traditional Chinese and Western Medicine (The Affiliated Hospital of Hunan Academy of Traditional Chinese Medicine), Changsha, Hunan, China

**Keywords:** fibular grafts, reconstructive surgery, orthopedic surgery, bibliometric study, research trends

## Abstract

**Background:**

Over the past few decades, fibular grafts have been widely utilized across 86 countries and regions globally for surgical reconstruction of various anatomical sites, including the mandible, upper extremities, lower extremities, spine, and in phalloplasty procedures. The present study aims to systematically investigate the developmental trajectory of fibular graft and identify research priorities for surgeons.

**Methods:**

A bibliometric analysis was conducted by searching the Web of Science Core Collection on April 12, 2024, for articles published between 2004 and 2023 on fibular grafting, using the query TS = (“graft” OR “transfer” OR “flap”) AND TS = (“fibular”). We included full-text English articles and reviews, and exclude documents that were not related to fibular grafting or were non-research-oriented publications. GraphPad Prism, CiteSpace, and VOSviewer analyzed publication trends and co-citation networks, providing insights into fibular grafting research.

**Results:**

A total of 2,884 fibular graft publications were analyzed. Out of 86 countries/regions, the United States and China stood out as the main contributors in terms of publication volume, while England had the highest citation rate per publication. The journals with the most publications and citations were *The Journal of Craniofacial Surgery* and *Plastic and Reconstructive Surgery,* respectively. Mark K. Wax had the most publications, while Hidalgo DA had the highest co-citation count. The most frequently occurring keywords were “reconstruction” and “mandibular reconstruction.” Co-citation reference clustering revealed a growing preference for vascularized fibular grafts over non-vascularized alternatives. The top 10 co-cited references were exclusively focused on mandibular reconstruction. Keyword bursts analysis showed that over the initial 20-year period, identified keywords fall into three main themes: graft design (e.g., osteoseptocutaneous flap, perforator flap), reconstruction areas (e.g., maxilla, extremity, ankle, spine and phalloplasty), and defect causes (e.g., pseudarthrosis, sarcoma, bone tumor). In particular, fibular grafts in phalloplasty represent an emerging trend among various anatomical reconstruction sites. In the last 5 years, there has been a notable rise in interest in 3D planning, virtual surgical planning, augmented reality, and reconstruction accuracy.

**Conclusion:**

The findings offer an in-depth overview of the landscape of fibular graft research, highlighting key contributors and emerging trends.

## 1 Introduction

Fibular grafts have become one of the most commonly used bone grafts for reconstructive surgery. Over the past two decades, they have been widely employed in the reconstruction of the mandible, repair of segmental defects, treatment of osteonecrosis of the femoral head, promotion of spine fusion (1), and as an alternative in phalloplasty (2). Beyond the basic use of strut-like fibular grafts to fill skeletal defects, several advanced harvesting techniques have emerged. Proximal fibular grafts, for example, allow reconstruction of the osteoarticular surface while preserving longitudinal growth and joint surface remodeling (3). Double-barrel fibular grafts, which use two struts to double the cross-sectional area, are preferred for the femur, proximal tibia, and spine over single-strut grafting (4). Cross-sectional area could also be augmented by combined fibular grafts with allografts/extracorporeal irradiated autografts. By threading the fibular graft through the medullary canal of the allograft, this technique leverages the mechanical strength of the allograft along with the healing potential and hypertrophic properties of the vascularized fibular graft (5). Additionally, fibular grafts can be harvested with soft tissue to repair composite tissue defects (6). Despite their extensive use in addressing conditions such as tumors (7), trauma, infectious non-union (8), and congenital pseudarthrosis (9), fibular grafts are associated with several potential complications. These include non-union or delayed union, stress fractures, and donor-site morbidities such as valgus ankle deformity and flexion contracture of the great toe (10).

Bibliometric analysis is a literature analysis method that examines the output and status of publications in a particular research field from both quantitative and qualitative perspectives (11). Through this analysis, we can obtain detailed information about authors, keywords, journals, countries, institutions, references, and more within the relevant research field. Bibliometric tools such as CiteSpace, VoSviewer, and the R package bibliometrix are commonly used to visualize the results of literature analysis. These tools have been widely applied in medical fields, particularly in reconstructive surgery (12) and orthopedic surgery (13). However, to date, no studies have applied bibliometric methods to analyze global research trends on fibular grafts. In light of this, the aims of this study were to: (1) Identify the current status of fibular grafts domain, including the distribution of annual outputs and the major contributors such as countries, institutions, and individuals. (2) Analyze the cooperation networks at the levels of countries, institutions, and authors. (3) Summarize the main research directions and hotspots. (4) Propose research frontiers and potential hotspots for the near future.

## 2 Methods

The Web of Science Core Collection (WoSCC) database is renowned for its superior accuracy in document type labeling, making it the preferred choice for conducting comprehensive literature analyses. Hence, on April 12, 2024, we initiated a search within WoSCC to identify all articles pertaining to fibular grafting published between January 1, 2004, and December 31, 2023. The search query employed was TS = (“graft” OR “transfer” OR “flap”) AND TS = (“fibular”).

**Figure 1:**
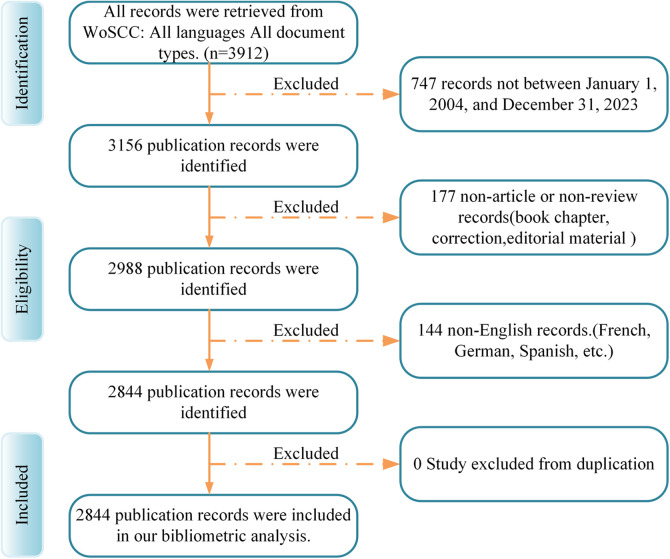
The search strategy used for the present bibliometric analysis. Search of the Web of Science database was conducted with the following approach: ([TS = (graft)] OR TS = (flap)) OR TS = (transfer) AND TS = (fibula). Studies published between January 1, 2004, and December 31, 2023, were considered for inclusion. Out of the initially identified 3,165 studies, 177 were excluded due to being book chapters, corrections, or editorial material, while 144 studies were excluded because they were not written in English. Ultimately, 2,844 studies remained and were included in the subsequent analyses. TS, topic search.

Our literature selection criteria were meticulously defined: (1) Inclusion of full-text publications directly related to fibular grafting; (2) Articles and review manuscripts written exclusively in English; (3) Articles published within the specified timeframe. Conversely, exclusion criteria were applied to filter out irrelevant content: (1) Articles not directly addressing fibular grafting; (2) Documents such as conference abstracts, news articles, brief reports, and other non-research-oriented publications. Upon selection, a plain text version of the identified papers was exported for further detailed analysis (Figure 1). This approach ensures that our study focuses specifically on scholarly contributions relevant to the field of fibular grafting, adhering strictly to high-quality research outputs within the specified parameters.

We used GraphPad Prism v8.0.2 to analyze yearly publication trends and national publication proportions in fibular grafting research. This software is effective for statistical analysis and visualizing data trends over time. Additionally, CiteSpace (version 6.2.4R, 64-bit advanced edition) and VOSviewer (version 1.6.18) were employed to construct and visualize co-citation networks, highlighting research achievements and trends within the field. CiteSpace, developed by Professor Chaomei Chen, is particularly useful for exploring new concepts and predicting future developments based on literature analysis. These tools collectively provide insights into the structure, evolution, and emerging trends of scientific literature related to fibular grafting.

## 3 Results

### 3.1 Distribution of articles by publication years

From January 1, 2004, to December 31, 2023, a total of 2,884 articles on fibular grafting were indexed in the WoSCC database. These comprised 2,644 research articles (94.31%) and 240 reviews (5.69%). The literature encompassed contributions from 86 countries and regions, involving 2,477 institutions and 10,393 authors.

**Figure 2:**
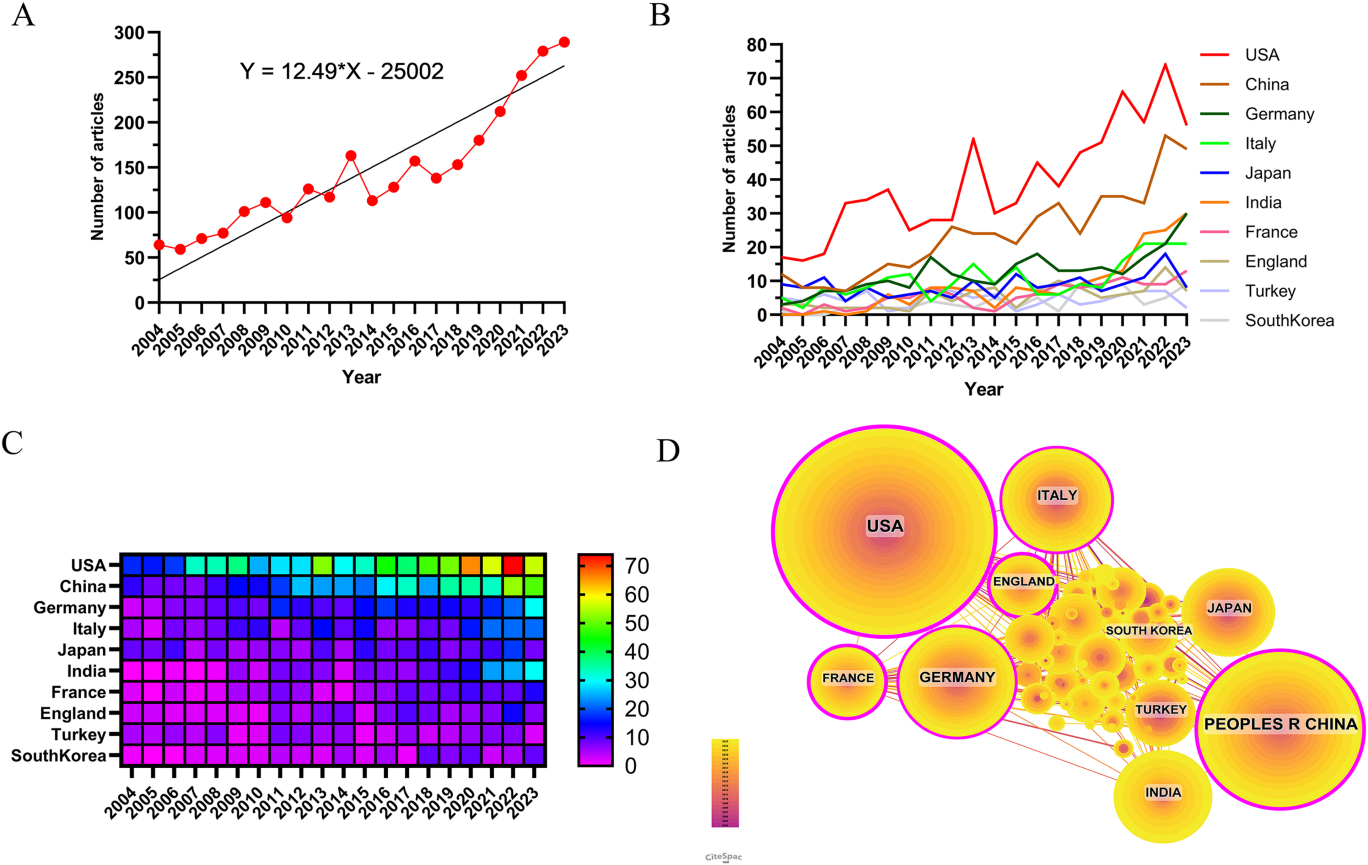
Trends in the number of publications and analysis of country/regions in fibular graft-related research. **(A)** The annual worldwide publication output. **(B)** Growth trends in the publication output from the top 10 countries. **(C)** The annual publication output from the top 10 countries. **(D)** Cooperation network of countries/regions. Each node in the graph symbolizes a country or region, with its size reflecting the volume of publications originating from that particular area. The thickness of the connections between nodes indicates the intensity of collaboration between the respective countries or regions. The shade of the node correlates with the timeline of the country's or region's initial publication, with darker shades representing earlier publications. Nodes covered by purple circles denote high centrality within the network.

Analyzing the publication trends over this period (Figure 2A), we observed three distinct stages. Initially, from 2004 to 2007, there was gradual growth with fewer than 100 papers published annually, indicating limited research attention in the field. The second stage, spanning from 2008 to 2016, witnessed a rapid increase in publications, signifying substantial development and heightened interest. Beyond 2017, there was a sustained upward trajectory in publications, culminating in a peak in 2023, highlighting continued growth and relevance in fibular grafting research.

### 3.2 Contribution of countries/regions and by institutions

Research on fibular grafting spans 86 countries and regions globally, as depicted in Figures 2B,C which illustrate the annual publication volumes of the top 10 countries over the past decade. The United States, China, Germany, Italy, and Japan emerge as the top contributors in this field, with the United States leading significantly with 27.25% of total publications.

In terms of citations (Table 1), the United States stands out with 15,528 citations, far exceeding other countries. Its citation/publication ratio of 19.76 ranks third globally, indicating high-quality research output. China follows closely in publication volume (479 papers) and citation count (7,360 times), with a slightly lower citation/publication ratio (15.37). Figure 2D reveals a collaborative network where the United States and China maintain strong ties. China collaborates closely with Japan, Turkey, and South Korea, while countries like Italy, Germany, and France also engage in collaborative efforts.

**Table 1:** The top 10 countries/regions with the greatest numbers of fibular graft relevant publications.

Rank	Country/region	Article counts	Centrality	Percentage (%)	Citation	Citation per publication
1	USA	786	0.46	27.25%	15,528	19.76
2	China	479	0.22	16.61%	7,360	15.37
3	Germany	249	0.17	8.63%	4,588	18.43
4	Italy	211	0.1	7.32%	4,236	20.08
5	Japan	171	0.03	5.93%	2,073	12.12
6	India	169	0.04	5.86%	1,108	6.56
7	France	114	0.2	3.95%	1,549	13.59
8	England	107	0.2	3.71%	3,124	29.20
9	Turkey	94	0.02	3.26%	1,035	11.01
10	South Korea	73	0.05	2.53%	716	9.81

Among 2,477 institutions contributing to fibular grafting research, the top 10 institutions by publication volume include 5 from the United States, 4 from China, and 1 from Germany (Table 2). The University of Texas System leads with 77 papers and 1,802 citations, averaging 23.40 citations per paper. Chang Gung Memorial Hospital follows closely with 74 papers and 2,335 citations (31.55 citations per paper), demonstrating high impact. The University of California System and Shanghai Jiao Tong University also feature prominently.

**Table 2:** The top 10 institution with the greatest numbers of fibular graft relevant publications.

Rank	Institution	Country	Number of studies	Total citations	Average citation
1	University of Texas System	USA	77	1,802	23.40
2	Chang Gung Memorial Hospital	China	74	2,335	31.55
3	University of California System	USA	63	1,574	24.98
4	Shanghai Jiao Tong University	China	57	908	15.93
5	Chang Gung University	China	56	1,557	27.80
6	Mayo Clinic	USA	55	915	16.64
7	Harvard University	USA	49	1,133	23.12
8	UTMD Anderson Cancer Center	USA	46	1,069	23.24
9	Peking University	China	46	471	10.24
10	Technical University of Munich	Germany	38	814	21.42

Further analysis indicates a tendency for both domestic and foreign institutions to collaborate more within their own countries. Strengthening international collaboration can enhance knowledge exchange and break down academic barriers in the field of fibular grafting.

### 3.3 Leading journal in this field

**Figure 3:**
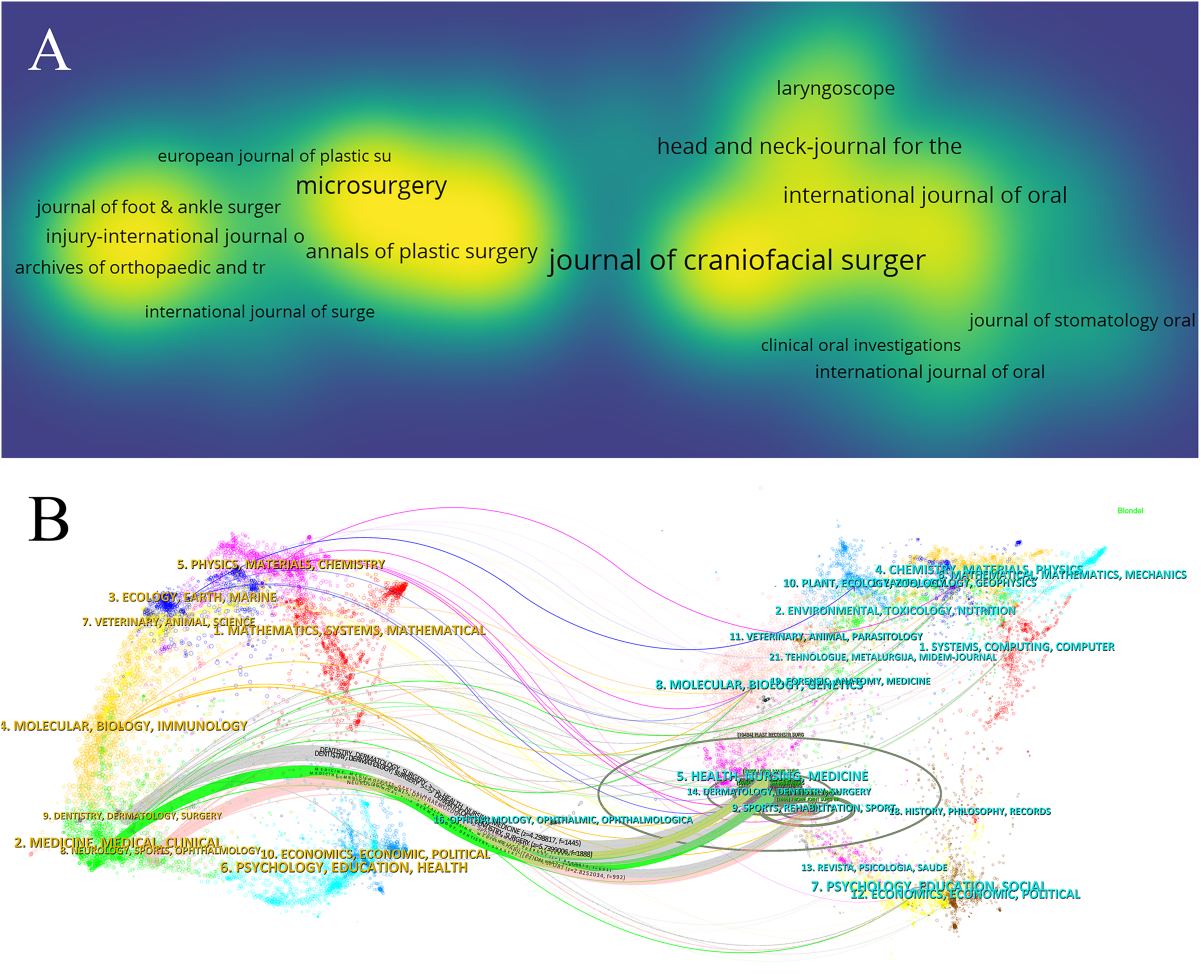
Analysis of journals and co-cited journals. **(A)** The density map of journals. **(B)** The dual-map overlay of journals related to fibular graft.

Tables 3, 4 provide insights into the top 10 journals with the highest publication output and most citations in the field of fibular grafting, respectively. The Journal of Craniofacial Surgery leads with 187 papers (6.48% of total publications), followed by Plastic and Reconstructive Surgery (137 papers, 4.75%), Journal of Oral and Maxillofacial Surgery (127 papers, 4.40%), and Microsurgery (127 papers, 4.40%). Notably, Plastic and Reconstructive Surgery holds the highest Impact Factor (IF) of 3.6 among these journals, with 60% classified in the Q1 or Q2 categories according to Journal Citation Reports (JCR). The density map (Figure 3A) visually illustrates the distribution of publications across these journals.

**Table 3:** The top 10 journals with the largest number of fibular graft-related research publications.

Rank	Journal	Article counts	Percentage (2,884)	IF	Quartile in category
1	*Journal of Craniofacial Surgery*	187	6.48%	0.9	Q4
2	*Plastic and Reconstructive Surgery*	137	4.75%	3.6	Q1
3	*Journal of Oral and Maxillofacial Surgery*	127	4.40%	1.9	Q4
4	*Microsurgery*	127	4.40%	2.1	Q2
5	*Journal of Plastic Reconstructive and Aesthetic Surgery*	94	3.26%	2.7	Q2
6	*Journal of Cranio-Maxillofacial Surgery*	91	3.16%	3.1	Q2
7	*Journal of Reconstructive Microsurgery*	89	3.09%	2.1	Q2
8	*International Journal of Oral and Maxillofacial Surgery*	72	2.50%	2.4	Q3
9	*Annals of Plastic Surgery*	68	2.36%	1.5	Q3
10	*Head and Neck-Journal for the Sciences and Specialties of the Head and Neck*	67	2.32%	2.9	Q1

**Table 4:** The top 10 journals with the largest co-citation of fibular graft-related research.

Rank	Author	Count	Location	Rank	Co-cited author	Citation
1	Heiland, M	27	Germany	1	Hidalgo DA	733
2	Wax, MK.	25	USA	2	Taylor GI	499
3	Hanasono, MM.	24	USA	3	Wei FC	440
4	Wei, FC	24	China	4	Urken ML	391
5	Rodriguez, ED.	23	USA	5	Cordeiro PG	281
6	Wolff, KD	23	Germany	6	Brown JS	261
7	Hoelzle, F	22	Germany	7	Hanasono, MM.	222
8	Blackwell, KE.	21	USA	8	Disa JJ	198
9	Matros, E	21	USA	9	Enneking WF	190
10	Rendenbach, C	20	USA	10	Jones NF	187

The influence of a journal is reflected in its co-citation frequency, demonstrating its impact within the scientific community. As shown in Table 4, Plastic and Reconstructive Surgery leads with 2,105 co-citations, followed by Journal of Oral and Maxillofacial Surgery (1,205 citations) and Microsurgery (1,057 citations). Among these top 10 journals by co-citations, Journal of Bone and Joint Surgery stands out with 908 citations and an Impact Factor (IF) of 5.3, also predominantly classified in Q1/Q2 journals (70%).

A dual-map overlay (Figure 3B) visually represents the thematic distribution of academic publications. It highlights six major citation pathways: research published in dentistry/dermatology/surgery journals primarily cites research in health/nursing/medicine, dermatology/dentistry/surgery, and sports/rehabilitation/sport fields. Similarly, research in medicine/medical/clinical fields is heavily cited by health/nursing/medicine and dermatology/dentistry/surgery journals. Moreover, neurology/sports/ophthalmology research is primarily cited by sports/rehabilitation/sport journals. These pathways underscore the interdisciplinary nature and collaborative influence across different domains in the field of fibular grafting research.

### 3.4 Authors and co-cited authors

**Figure 4:**
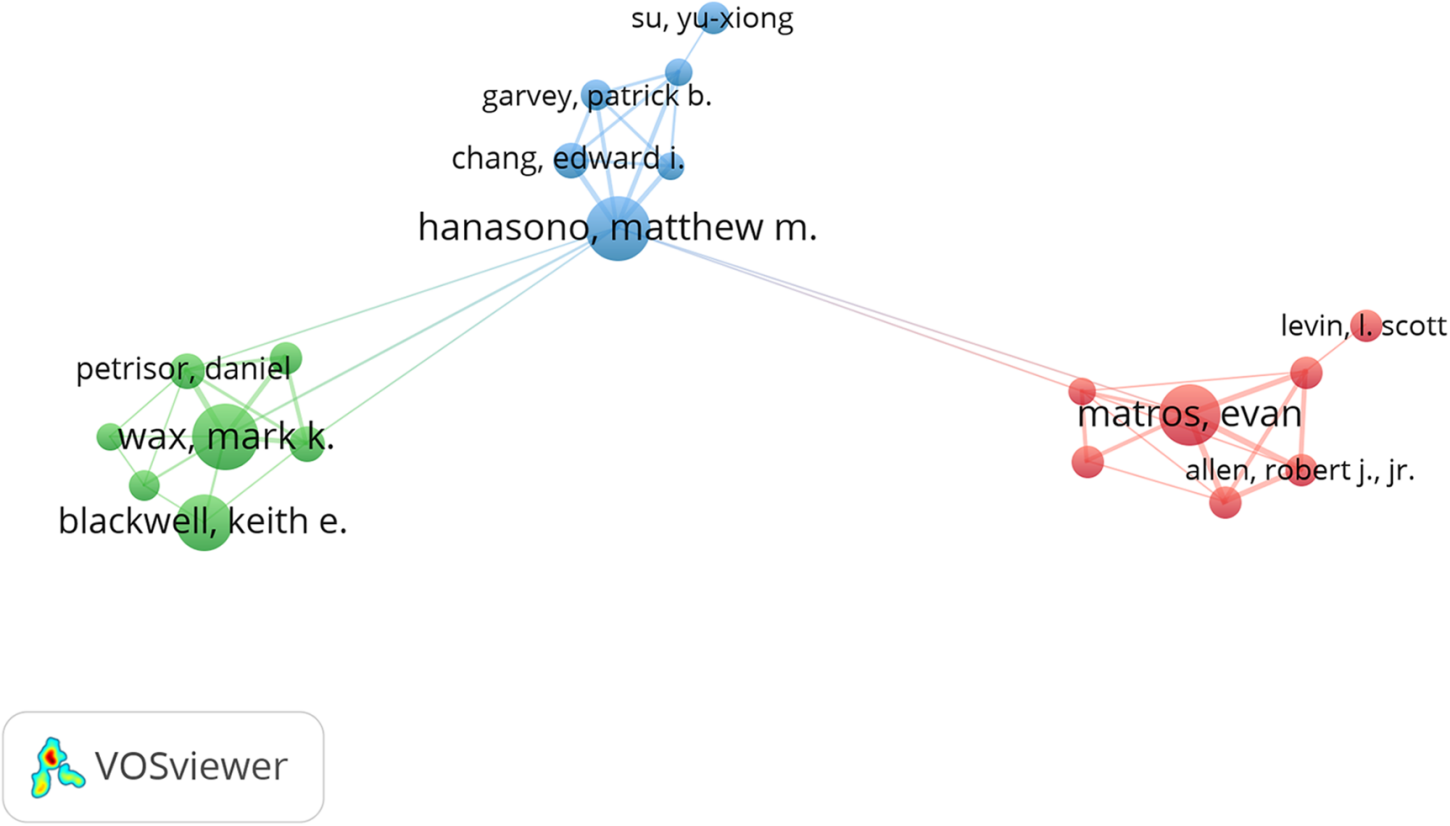
The visualization of cited authors on research of fibular graft.

Table 5 showcases the top 10 authors who have contributed significantly to literature on fibular grafting. Collectively, these authors have authored 230 papers, representing 7.97% of all publications in this field. Max Heiland leads with 27 papers, followed closely by Mark K. Wax with 25 papers and Matthew M. Hanasono with 24 papers. Analysis further reveals that 6 out of these top 10 authors are based in the United States, 3 in Germany, and 1 in China. Visualized in Figure 4 using CiteSpace, the network among authors shows clustering into three modules. Notably, Mark K. Wax, Matthew M. Hanasono, and Evan Matros emerge as key collaborators within and across these modules. Table 5 highlight the top 10 authors with the highest co-citations and citations. Fifty-four authors have been cited more than 20 times, underscoring the significant impact and influence of their research. The largest nodes in the network represent authors with substantial co-citations, such as Hidalgo Da (733 citations), Taylor GI (499 citations), and Wei FC (440 citations). These findings underscore the prominence of these researchers in the field of fibular grafting.

**Table 5:** Top 10 authors and co-cited authors on research of fibula grafts.

Rank	Cited Journal	Co-citation	IF (2,022)	Quartile in category
1	*Plastic and Reconstructive Surgery*	2,105	3.6	Q1
2	*Journal of Oral and Maxillofacial Surgery*	1,205	1.9	Q4
3	*Microsurgery*	1,057	2.1	Q2
4	*Head & Neck: Journal for the Sciences and Specialties of the Head and Neck*	957	2.9	Q1
5	*Journal of Cranio-Maxillofacial Surgery*	949	3.1	Q2
6	*Journal of Reconstructive Microsurgery*	938	2.1	Q2
7	*International Journal of Oral and Maxillofacial Surgery*	917	2.4	Q3
8	*Journal of Bone and Joint Surgery, American Volume*	908	5.3	Q1
9	*Annals of Plastic Surgery*	902	1.5	Q3
10	*Clinical Orthopaedics and Related Research*	866	4.3	Q1

### 3.5 Co-cited references

The top 10 most co-cited articles, as listed in Table 6, shed light on significant contributions to the field. The most cited article, “Computer-assisted design and rapid prototype modeling in microvascular mandible reconstruction” published in Laryngoscope, highlights the benefits of computer-assisted design in reducing surgical time and improving the accuracy of mandibular reconstruction (14). Following closely is “A new classification for mandibular defects after oncological resection,” published in The Lancet Oncology, which proposes a novel classification system for guiding mandibular reconstruction efforts (15). Ranked third is “Improved operative efficiency of free fibula flap mandible reconstruction with patient-specific, computer-guided preoperative planning,” published in Head and Neck (16), emphasizing efficiency gains through computer-assisted planning in fibular graft-based mandibular reconstruction. These top three articles exclusively focus on mandibular reconstruction, underscoring the dominant use of fibular grafts in this context. One possible explanation for this focus is the availability of alternative techniques for reconstructing other body parts, such as limb reconstruction and femoral head revascularization, where iliac crest flaps are often preferred (10). Moreover, in penile reconstruction, the forearm radial flap is favored over fibular grafts due to its sensory advantages (17).

**Table 6:** Top 10 co-cited references on research of fibular grafts.

Rank	Title	Journal	Author(s)	Total citations
1	Computer-assisted design and rapid prototype modeling in microvascular mandible reconstruction	*Laryngoscope*	Hanasono MM	43
2	A new classification for mandibular defects after oncological resection	*Lancet Oncology*	Brown JS	43
3	Improved operative efficiency of free fibula flap mandible reconstruction with patient-specific, computer-guided preoperative planning	*Head And Neck-Journal For The Sciences And Specialties Of The Head And Neck*	Toto JM	41
4	The Accuracy of Virtual Surgical Planning in Free Fibula Mandibular Reconstruction: Comparison of Planned and Final Results	*Journal of Oral and Maxillofacial Surgery*	Roser SM	40
5	Mandibular Reconstruction Using Computer-Aided Design and Computer-Aided Manufacturing: An Analysis of Surgical Results	*Journal of Oral and Maxillofacial Surgery*	Foley BD	39
6	Functional Outcomes of Virtually Planned Free Fibula Flap Reconstruction of the Mandible	*Plastic and Reconstructive Surgery*	Avraham T	39
7	Use of Computer-Aided Design and Computer-Aided Manufacturing to Produce Orthognathically Ideal Surgical Outcomes: A Paradigm Shift in Head and Neck Reconstruction	*Journal of Oral and Maxillofacial Surgery*	Hirsch DL	36
8	Early and late complications in the reconstructed mandible with free fibula flaps	*Journal of Surgical Oncology*	Van Gemert JTM	36
9	Survival of dental implants placed in vascularised fibula free flaps after jaw reconstruction	*Journal of Cranio-Maxillofacial Surgery*	Attia S	35
10	Use of Virtual Surgery and Stereolithography-Guided Osteotomy for Mandibular Reconstruction with the Free Fibula	*Plastic and Reconstructive Surgery*	Antony AK	35

**Figure 5:**
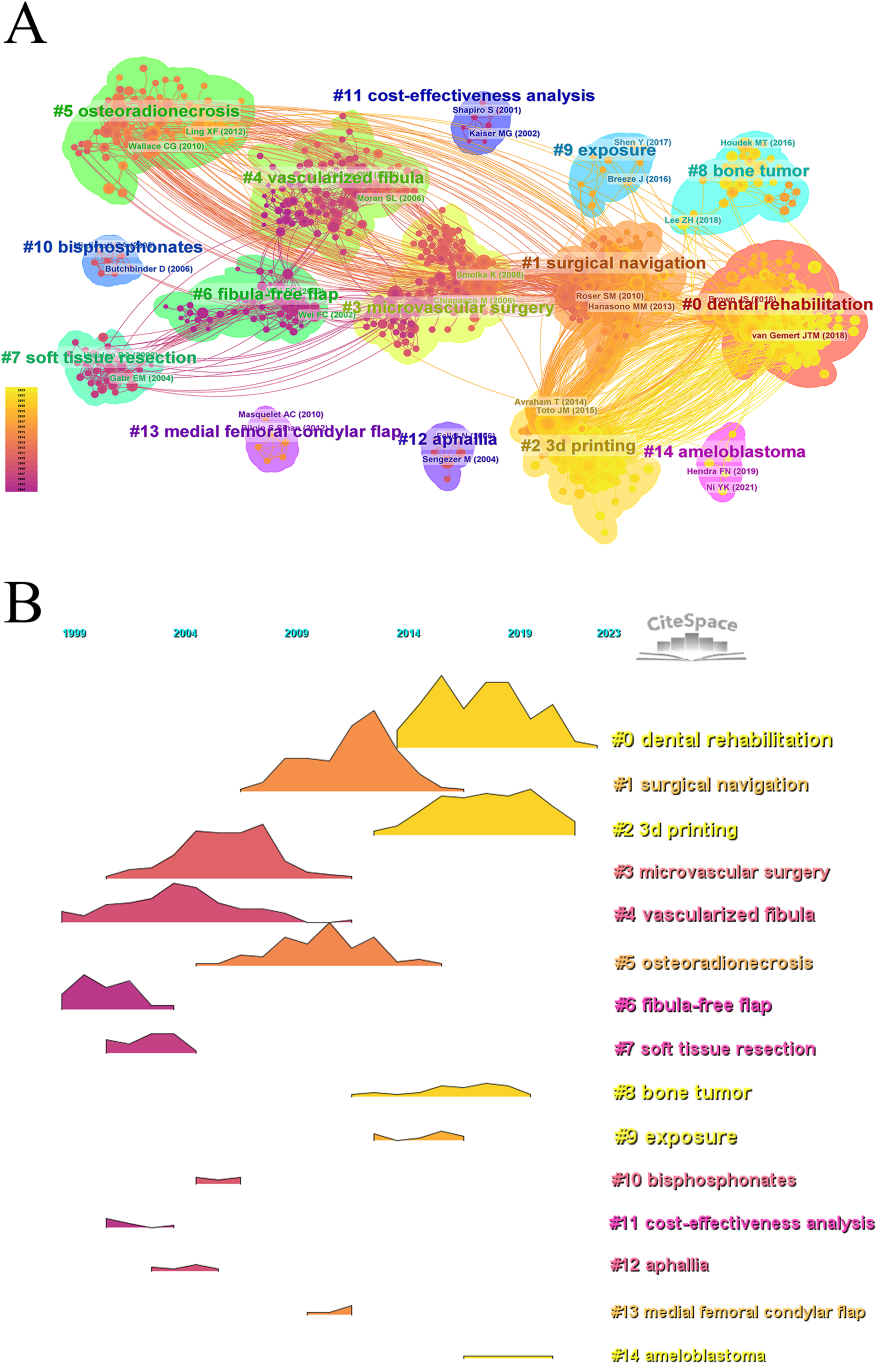
Visualizing co-cited literatures on research of fibular graft, by showing clustering **(A)** and volcano diagram **(B)**.

We conducted co-citation reference clustering and temporal clustering analysis (Figures 5A,B). Our findings reveal that microvascular surgery (cluster 3), vascularized fibula (cluster 4), fibula free flap (cluster 6), soft tissue reconstruction (cluster 7), and cost-effectiveness analysis (cluster 11) were early research hotspots. Moving into mid-term research, surgical navigation (cluster 1), osteoradionecrosis (cluster 6), bisphosphonates (cluster 10), aphallia (cluster 12), and medical femoral condylar flap (cluster 13) gained prominence. In addition, topics such as dental rehabilitation (cluster 0), 3D printing (cluster 2), bone tumors (cluster 8), exposure (cluster 9), and ameloblastoma (cluster 14) emerged as significant trends in the field over time. These clusters illustrate the evolving focus areas and research trends within fibular graft applications.

**Figure 6:**
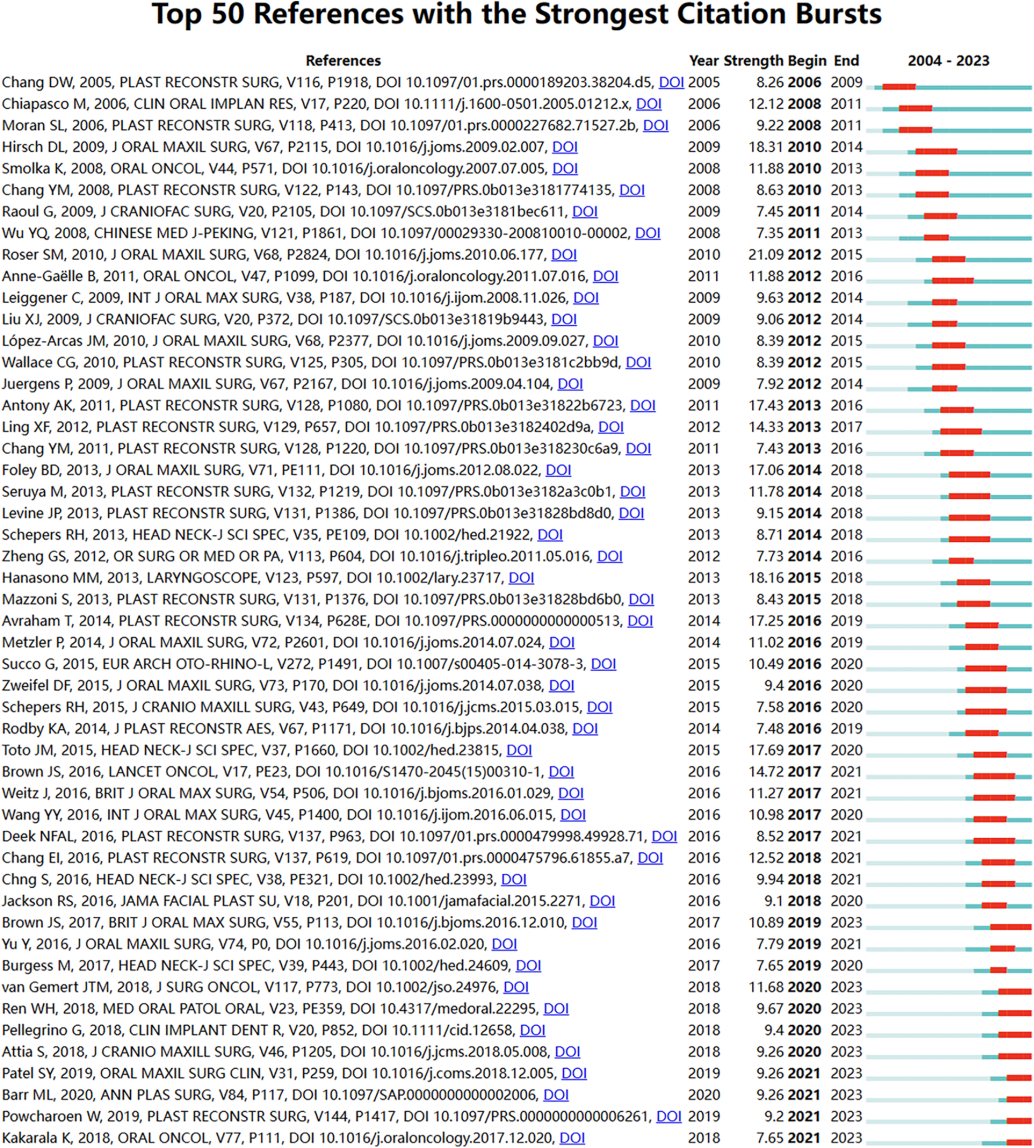
Top 50 references with the strongest citation bursts. A red bar indicates high citations in that year. The blue bars were references cited less frequently.

Through CiteSpace, we investigated the citation bursts of references in the field of fibular grafting and identified the top 50 most influential citations. The most cited reference, with 21.09 citations, is the article titled “The Accuracy of Virtual Surgical Planning in Free Fibula Mandibular Reconstruction: Comparison of Planned and Final Results,” published in the Journal of Oral and Maxillofacial Surgery. These references span publications from 2004 to 2023 and have consistently garnered significant citations over the past two decades. Notably, nine of these papers are currently experiencing a peak in citations (Figure 6), indicating sustained and increasing interest in fibular grafting moving forward. This highlights the ongoing relevance and importance of research in this area.

### 3.6 Keywords analysis

**Figure 7:**
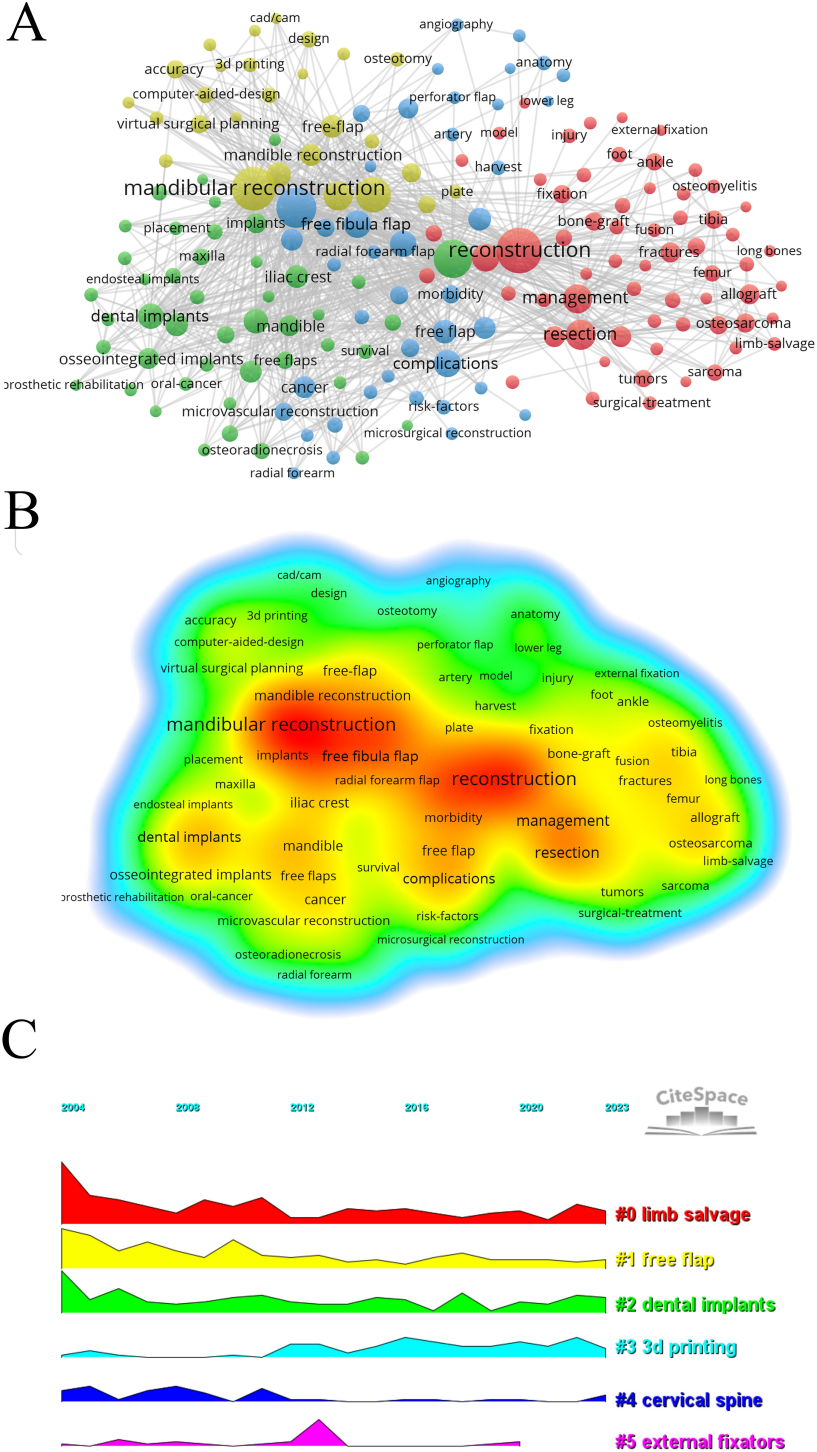
The visualization of keywords on research of fibular graft. **(A)** Network visualization of keywords in research on fibular graft mapped four distinct clusters consisting of nodes with the same color. **(B)** Term density map of keywords in the field of fibular graft in the WoSCC database. The heatmap illustrates the frequency of keywords through varying shades of color; vibrant red indicates high-frequency keyword, while cool blue represents low-frequency keywords. **(C)** The volcano plot showing timeline view of cluster analysis of keywords.

By analyzing keywords, we can gain rapid insights into the current state and future directions of a field. Based on co-occurrence data visualized in VOSviewer, the most prominent keywords include “reconstruction” (615 occurrences), followed closely by “mandibular reconstruction” (553), “head” (447), “defects” (405), and “surgery” (332) (Supplementary Table S1, Figures 7A,B). After filtering out less relevant keywords, we constructed a network comprising 177 keywords that appeared at least 23 times, revealing four distinct clusters. The first cluster (red) includes 63 keywords with high frequencies for terms like reconstruction, management, salvage, bone graft, fractures, resection, ankle, knee, system, fixation, repair, transport, extremity, internal fixation, femur, foot, fusion, injury, model, replacement, and stability. The second cluster (green) features 45 keywords, emphasizing quality of life, maxilla, implants, scapula, jaws, oral cancer, dental implants, mandible, free flaps, patient, radiotherapy, tissue, survival, and midface. The third cluster (blue) comprises 42 keywords focusing on artery, anatomy, angiography, complication, failure, donor site morbidity, harvest, lower leg, muscle, neck reconstruction, perforator flap, risk factor, and vessels. The fourth cluster (yellow) contains 27 keywords with key terms such as mandibular reconstruction, outcome, surgery, defect, classification, plates, design, osteotomy, 3D printing, accuracy, navigation, simulation, computer-assisted surgery, technology, and virtual planning.

Using CiteSpace, we generated a volcano map (Figure 7C) that illustrates the evolution of research hotspots over time. This map highlights the temporal distribution of five keyword clusters relevant to fibular grafts: free flap, dental implants, 3D printing, cervical spine, and external fixators.

**Figure 8:**
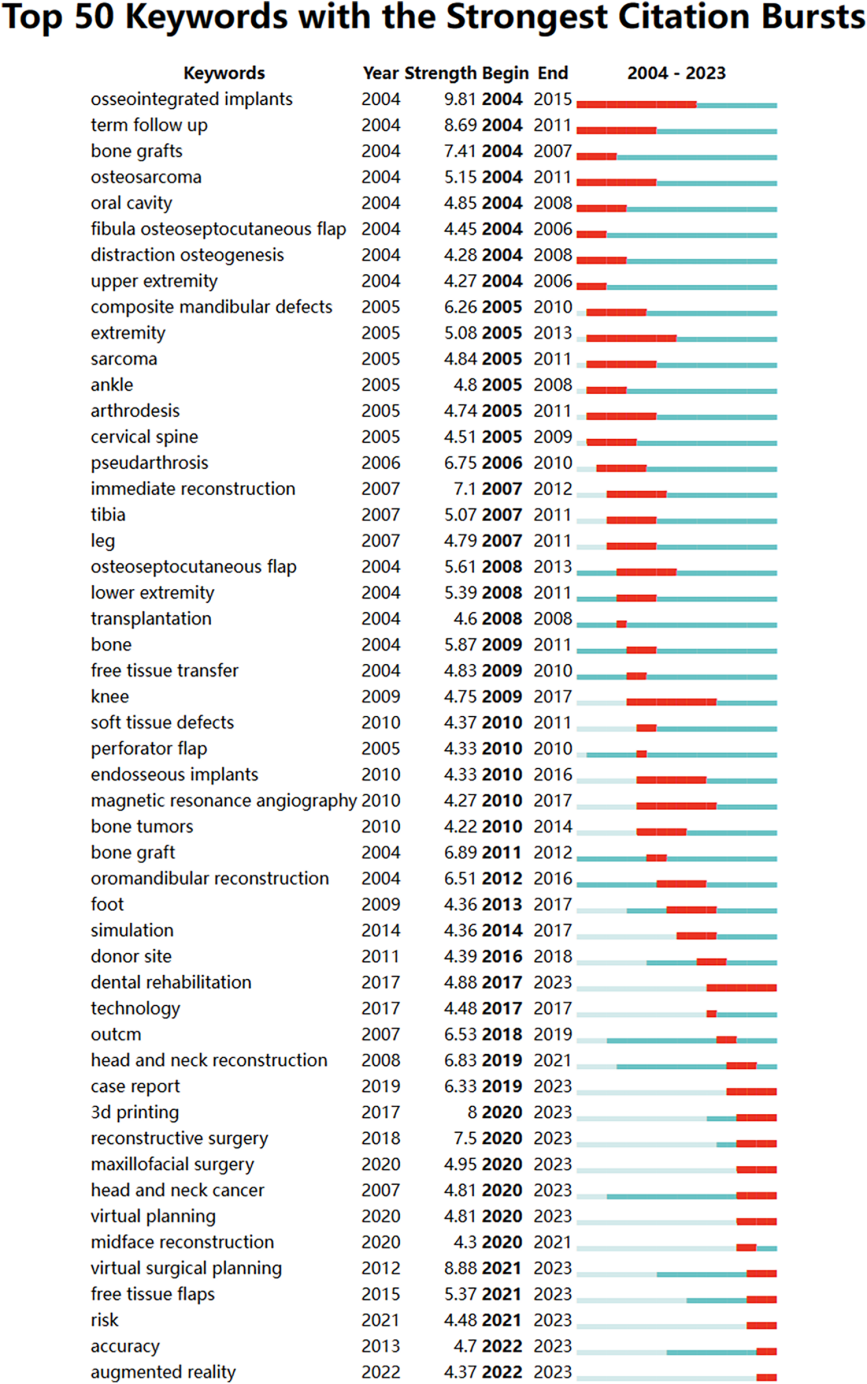
Top 50 keywords with the strongest citation bursts. A red bar indicates high citations in that year. The blue bars were references cited less frequently.

In this study, the CiteSpace Bursts detection algorithm was utilized to generate a keyword hotspot evolution map spanning from 2004 to 2023 in the field of fibular grafts on the Web of Science, specifically identifying keyword bursts. A total of 756 keyword bursts were identified. Concurrently, the study also identified the top 50 keywords in fibular grafts. The burst intensity and duration of hotspots are depicted in Figure 8, where red line segments indicate the start and end years of each burst period. In the initial phase of the past two decades, the identified keywords can be broadly categorized into three themes: design of grafts (such as osteoseptocutaneous flap, perforator flap), areas of reconstruction (including maxilla, extremity, ankle, spine), and reasons for defects (such as pseudarthrosis, sarcoma, bone tumor). In recent years, particularly over the last 5 years, there has been a notable increase in attention towards 3D planning, virtual surgical planning, augmented reality, and the accuracy of reconstruction. This focus aligns closely with a surge in keywords related to mandibular reconstruction, notably coinciding with the keyword burst “dental rehabilitation” observed in recent years.

## 4 Discussion

The present study uses bibliometric analysis to quantitatively and visually demonstrate research trends and changes in the field of fibular grafting. With the annual publication volume keeps increasing, fibular grafting has become a prominent topic in reconstructive surgery. Globally, the USA and China have made the greatest impact on fibular graft research, as inferred from publication volume, total citations, and the growth in the number of publications, although England has the highest citation per publication. Institutional analysis further confirms that the major contributions come from the USA and China, with 9 out of the top 10 institutions located in these two countries. Author and co-cited author analyses also reveal that among the top ten authors, 6 are from the United States, 3 are from Germany, and 1 is from China. Notably, in the author analysis, Max Heiland and Mark K. Wax, who have published a higher number of studies, as well as Hidalgo DA, Taylor GI, and Wei FC, who have the highest citation counts, generally possess greater representation and a higher reputation in the field.

When choosing journals to publish their research results, researchers typically seek journals that are relevant to their topic and can reach a large audience of interested readers (18). Consequently, much of the research on fibular grafts has been published in journals focused on plastic or reconstructive surgery. For instance, 137 papers have been published in Plastic and Reconstructive Surgery, 127 in Microsurgery, and 94 in Journal of Plastic, Reconstructive and Aesthetic Surgery. Interestingly, the journal with the highest number of publications is the Journal of Craniofacial Surgery (ranked 1st), which focuses more exclusively on mandibular surgery. Similarly, among the top 10 leading journals by publication volume, there are four other journals specializing in mandibular surgery: the Journal of Oral and Maxillofacial Surgery, the Journal of Cranio-Maxillofacial Surgery, the International Journal of Oral and Maxillofacial Surgery, and Head and Neck. This aligns with the data showing that fibular-graft-related papers on mandibular reconstruction outnumber those in other orthopedic surgeries, such as upper or lower limb, spine, and phalloplasty. Furthermore, the top 10 co-cited papers in the field of fibular grafts are exclusively related to mandibular reconstruction. However, considering the quality of publications, the journal with the highest citation or co-citation count is Plastic and Reconstructive Surgery, which more broadly covers reconstructive surgery rather than focusing solely on maxillofacial reconstruction.

In fact, fibular grafts are commonly employed in limb reconstruction (19), spinal reconstruction (20, 21), and phalloplasty (22). The fibular diaphysis is particularly valuable for addressing segmental defects due to its strut-like shape, while the fibular proximal allow joint remodeling and epiphyseal growth post-transfer. Vascularized fibular grafts are utilized in treating spine non-unions due to their ability to integrate well with spine and to support, underscored by “cervical spine” as a prominent keyword cluster in temporal distribution (Figure 7C). Further citation burst reveals that this trend began in 2005 and peaked around 2009 (Figure 8). Additionally, fibular grafts are used in phalloplasty to provide the necessary rigidity for neo-phalloplasty, crucial for deep penetration during sexual intercourse, highlighted by “aphallia” in temporal clustering analysis (Figures 5A,B). However, the keyword analysis did not extensively cover the most influential research in various reconstructive fields, as the top 10 co-cited studies exclusively focused on mandibular reconstruction. To enhance the identification of high-quality findings in fibular graft research, the authors of present study have compiled a list of the most cited articles categorized by different reconstructive goals (Supplementary Table S1). This focused approach helps surgeons in diverse specialties to pinpoint relevant and top-tier research specific to their respective fields.

Regarding fibular transfer, it's important to note that fibular grafts can be either vascularized or non-vascularized. Vascularized fibular grafts necessitate microvascular anastomosis, a procedure that enhances blood perfusion and significantly improves the chances of flap survival. First described by G.I. Taylor in 1975 for leg reconstruction, vascularized fibular grafts have now become a standard procedure in reconstructive surgery (23). Keyword analysis ranks “microvascular surgery” and “vascularized fibula” in the top 4 and top 5, respectively (Figure 5), indicating growing acceptance of vascularized over non-vascularized fibular grafts.

In co-citation reference clustering, “osteoradionecrosis” emerges as one of the top 15 keywords, highlighting the extensive use of fibular grafts in managing osteonecrosis. This popularity arises from the perceived benefit of enhancing blood perfusion when the flap is vascularized by pedicles. Despite this, there remains ongoing interest in utilizing non-vascularized fibular grafts for reconstructing diaphyseal defects and treating osteonecrosis (24, 25). Comparative studies directly comparing these two techniques would be highly valuable in delineating the specific advantages offered by vascularization. Unfortunately, there is a notable scarcity of comparative studies directly comparing vascularized and non-vascularized fibular grafts. Only two studies have reported on the advantages of vascularized fibular grafts, highlighting improvements in clinical scoring outcomes (26) or radiological outcomes (27).

The evolving research trends in fibular graft research was also revealed, by citation burst studies. Notably, augmented reality, virtual surgical planning, and 3D planning have seen significant citation bursts (Figure 7). Those virtual reality techniques, particularly in mandibular reconstruction, have been recognized as crucial, with papers utilizing virtual reality exclusively focused on this field. Within the realm of fibular-graft-based mandibular reconstruction, three of the top five most cited original articles explore the feasibility and efficacy of computer-assisted design and virtual surgical planning (refer to Supplementary Table S1), with Roser's seminal 2010 study garnering the highest citations per year (19.13/year) (28). These findings collectively underscore surgeons' heightened emphasis on the challenges and benefits associated with precisely applying fibular grafts in mandibular reconstruction (29). However, the challenges specific to applying fibular grafts in reconstructing other anatomical areas are not adequately reflected in current keyword burst analysis. While using fibular grafts for reconstruction across anatomical sites presents common challenges such as vascular compromise and donor-site morbidities (30, 31), specific challenges vary significantly. For instance, lower limb reconstruction using fibular grafts may involve higher risk of stress fractures due to weight-bearing (32), while pediatric cases require growth potential of fibular graft (33). Joint-related reconstructions involving the distal radius, humerus, or femoral head pose specific challenges related to joint remodeling (34, 35). The lack of relevant keywords in citation bursts, indicating a scarcity of research addressing these specific challenges, may be attributed to the lower demands for personalization and precision in these anatomical areas, as well as lower technical sensitivity compared to mandibular reconstruction.

One limitation of this manuscript is the restriction of the literature to English-language publications and the exclusive use of the Web of Science Core Collection (WoSCC) database. This approach may introduce potential publication bias, as non-English studies and research indexed in other databases, such as PubMed or Scopus, are excluded. Consequently, this may lead to an incomplete representation of the available evidence, limiting the diversity and comprehensiveness of the data included in the study. Another limitation of the current study lies in the search strategy: the diversity encompassed by the “fibular graft” category. While the predominant use of fibular grafts involves bone reconstruction, they are also employed as bone flaps or combined with skin paddles to address both osteo- and soft-tissue defects. Keywords such as “fibula osteoseptocutaneous flap” (36) “composite mandibular defects” “osteoseptocutaneous flap” and “soft tissue defects” prominently feature among the top 50 keywords with the strongest citation bursts. This trend was particularly notable from 2004 to 2010, coinciding with advancements in perforator flap techniques and the growing acceptance of composite tissue transfer approaches. However, other categories of fibular grafts, such as proximal fibular grafts, double-barrel techniques, and onlay/hybrid fibular grafting, are inadequately represented in both keyword and reference analyses. To comprehensively capture the diversity of fibular graft applications, a nuanced understanding of these graft types is crucial. This knowledge not only addresses the limitations of general bibliometric analysis but also enhances our grasp of the evolving landscape of fibular graft research and applications.

In conclusion, the bibliometric analysis underscores global trends in fibular graft research, revealing an expanding interdisciplinary field involving various reconstructive teams specializing in mandibular, upper and lower extremity, spine, and phalloplasty surgeries. Leading contributions originate from the USA and China, with impactful publications prominently featured in journal Plastic and Reconstructive Surgery. The University of Texas System stands out as the most influential institution, while Mark K. Wax and Hidalgo DA lead in terms of publication volume and co-citations, respectively. Key current research focuses include advancements in computer-assisted surgery and improving the precision of mandibular reconstruction.

## Data Availability

The raw data supporting the conclusions of this article will be made available by the authors, without undue reservation.
