# Supplementary material for: Fibular grafts in global reconstructive surgery: a bibliometric analysis
Source: Front Surg. 2024 Nov 18;11:1479878. doi: 10.3389/fsurg.2024.1479878 (PMC11609196; doi:10.3389/fsurg.2024.1479878)
Supplement: Supplementary file 1 [file Table1.docx]

**Supplementary Table S1**. The top 5 most cited original research studies in each relevant research field pertaining to fibular-graft-based reconstruction were identified using tailored search queries spanning from 1945 to 2024. Only articles classified as primary research were included, following a rigorous manual screening process to exclude reviews and non-relevant publications. Subsequently, the highest cited studies meeting these stringent criteria were compiled and presented in table.

| Research filed  [search term] | Search formula | Number of publications  (Article only) | Rank | Authors | Title (DOI) | Journal | Year | Total Citations  (total citation/year) |
| --- | --- | --- | --- | --- | --- | --- | --- | --- |
| Mandibular reconstruction | TS=('mandibular' OR 'mandible') AND TS=('graft' OR 'transfer' OR 'flap') AND TS=('fibular') | 649 | 1 | Wei FC, Seah CS, Tsai YC, Liu SJ, Tsai MS | Fibula osteoseptocutaneous flap for reconstruction of composite mandibular defects  (10.1097/00006534-199402000-00009) | Plast Reconstr Surg | 1994 | [320](https://www.webofscience.com/wos/woscc/citing-summary/3202995?type=refid)  (10.32) |
|  |  |  | 2 | Roser, SM; Ramachandra, S; Blair, H; Grist, W; Carlson, GW; Christensen, AM; Weimer, KA; Steed, MB | The Accuracy of Virtual Surgical Planning in Free Fibula Mandibular Reconstruction: Comparison of Planned and Final Results  (10.1016/j.joms.2010.06.177) | JOURNAL OF ORAL AND MAXILLOFACIAL SURGERY | 2010 | [287](https://www.webofscience.com/wos/woscc/citing-summary/339277629?type=refid)  (19.13) |
|  |  |  | 3 | Hanasono, Matthew M.; Skoracki, Roman J. | Computer-assisted design and rapid prototype modeling in microvascular mandible reconstruction (10.1002/lary.23717) | LARYNGOSCOPE | 2013 | [190](https://www.webofscience.com/wos/woscc/citing-summary/445103439?type=refid)  (15.83) |
|  |  |  | 4 | Wei, FC; Celik, N; Chen, HC; Cheng, MH; Huang, WC | Combined anterolateral thigh flap and vascularized fibula osteoseptocutaneous flap in reconstruction of extensive composite mandibular defects  (10.1097/00006534-200201000-00008) | PLASTIC AND RECONSTRUCTIVE SURGERY | 2002 | [164](https://www.webofscience.com/wos/woscc/citing-summary/26404676?type=refid)  (7.13) |
|  |  |  | 5 | Antony, Anuja K.; Chen, Wei F.; Kolokythas, Antonia; Weimer, Katherine A.; Cohen, Mimis N. | Use of virtual surgery and stereolithography-guided osteotomy for mandibular reconstruction with the free fibula (10.1097/PRS.0b013e31822b6723) | PLASTIC AND RECONSTRUCTIVE SURGERY | 2011 | [163](https://www.webofscience.com/wos/woscc/citing-summary/424052654?type=refid)  (11.64) |
| Upper extremity reconstruction | TS=('radius' OR 'humerus' OR 'upper extremities') AND TS=('graft' OR 'transfer' OR 'flap') AND TS=('fibular') | 272 | 1 | O'Connor MI, Sim FH, Chao EY | Limb salvage for neoplasms of the shoulder girdle - Intermediate reconstructive and functional results  (10.2106/00004623-199612000-00011) | J Bone Joint Surg Am | 1996 | 145  (5) |
|  |  |  | 2 | Jupiter JB, Gerhard HJ, Guerrero J, Nunley JA, Levin LS. | Treatment of segmental defects of the radius with use of the vascularized osteoseptocutaneous fibular autogenous graft  (10.2106/00004623-199704000-00009) | J Bone Joint Surg Am | 1997 | 90  (3.21) |
|  |  |  | 3 | Osterhoff G, Baumgartner D, Favre P, Wanner GA, Gerber H, Simmen HP, Werner CM. | Medial support by fibula bone graft in angular stable plate fixation of proximal humeral fractures: an in vitro study with synthetic bone  (10.1016/j.jse.2010.10.040) | J Shoulder Elbow Surg | 2011 | 85  (6.07) |
|  |  |  | 4 | Cheng CY, Shih HN, Hsu KY, Hsu RW | Treatment of giant cell tumor of the distal radius  (10.1097/00003086-200102000-00026) | Clin Orthop Relat Res | 2001 | 83  (3.46) |
|  |  |  | 5 | Jain AK, Sinha S | Infected nonunion of the long bones  (10.1097/01.blo.0000152868.29134.92) | Clin Orthop Relat Res | 2005 | 82  (4.1) |
| Lower extremity reconstruction | TS=('hip' OR 'tibial' OR 'lower extremities') AND TS=('graft' OR 'transfer' OR 'flap') AND TS=('fibular') AND TS=('reconstruction) | 314 | 1 | Yazar S, Lin CH, Wei FC | One-stage reconstruction of composite bone and soft-tissue defects in traumatic lower extremities  (10.1097/01.prs.0000138811.88807.65) | Plast Reconstr Surg | 2004 | 228  (10.86) |
|  |  |  | 2 | Song HR, Cho SH, Koo KH, Jeong ST, Park YJ, Ko JH | Tibial bone defects treated by internal bone transport using the Ilizarov method  (10.1007/s002640050263) | Int Orthop | 1998 | 88  (3.26) |
|  |  |  | 3 | Jain AK, Sinha S | Infected nonunion of the long bones  (10.1097/01.blo.0000152868.29134.92) | Clin Orthop Relat Res | 2005 | 82  (4.1) |
|  |  |  | 4 | Cavadas PC | Arteriovenous vascular loops in free flap reconstruction of the extremities  (10.1097/01.prs.0000297634.53915.e5) | Plast Reconstr Surg | 2008 | 72  (4.24) |
|  |  |  | 5 | Gonzalez MH, Tarandy DI, Troy D, Phillips D, Weinzweig N | Free tissue coverage of chronic traumatic wounds of the lower leg  (10.1097/00006534-200202000-00028) | Plast Reconstr Surg | 2002 | 71  (3.09) |
| Spine | TS=('spine' OR 'spinal') AND TS=('graft' OR 'transfer' OR 'flap') AND TS=('fibular') AND TS=('reconstruction) | 88 | 1 | Mobbs, RJ, Coughlan, M, Thompson, R, Sutterlin, CE, Phan, K | The utility of 3D printing for surgical planning and patient-specific implant design for complex spinal pathologies: case report | J Neurosurg Spine | 2017 | 131  (16.38) |
|  |  |  | 2 | Hilibrand AS, Fye MA, Emery SE, Palumbo MA, Bohlman HH | Impact of smoking on the outcome of anterior cervical arthrodesis with interbody or strut-grafting  (10.2106/00004623-200105000-00004) | J Bone Joint Surg Am | 2001 | 123  (5.13) |
|  |  |  | 3 | Jin D, Qu D, Chen J, Zhang H | One-stage anterior interbody autografting and instrumentation in primary surgical management of thoracolumbar spinal tuberculosis  (10.1007/s00586-003-0661-5) | Eur Spine J | 2004 | 100  (4.76) |
|  |  |  | 4 | Rajasekaran S, Vijay K, Shetty AP | Single-stage closing-opening wedge osteotomy of spine to correct severe post-tubercular kyphotic deformities of the spine: a 3-year follow-up of 17 patients  (10.1007/s00586-009-1234-z) | Eur Spine J | 2010 | 73  (4.87) |
|  |  |  | 5 | Dietze DD Jr, Fessler RG, Jacob RP | Primary reconstruction for spinal infections  (10.3171/jns.1997.86.6.0981) | J Neurosurg | 1997 | 72  (2.57) |
| phalloplasty | TS=('phalloplasty' OR 'neophallus ' OR 'penile') AND TS=('graft' OR 'transfer' OR 'flap') AND TS=('fibular') | 15 articles and 8 review | 1 | Sengezer M, Oztürk S, Deveci M, Odabaşi Z | Long-term follow-up of total penile reconstruction with sensate osteocutaneous free fibula flap in 18 biological male patients  (10.1097/01.prs.0000131883.27191.86) | Plast Reconstr Surg | 2004 | 61  (2.9) |
